# 49. Effects of Maternal SARS-CoV-2 Infection on Neonatal Discharge Planning and Care: Exacerbation of Racial and Ethnic Healthcare Disparities

**DOI:** 10.1093/ofid/ofab466.049

**Published:** 2021-12-04

**Authors:** Jennifer Jubulis, Amanda Goddard, Sarah Dibrigida, Carol A McCarthy

**Affiliations:** Maine Medical Center, Portland, Maine

## Abstract

**Background:**

SARS-CoV-2 has exacerbated healthcare disparities. Maine’s population of 1.3 million is comprised of only 6% Black, Indigenous, People of Color (BIPOC); however, statewide 18% of SARS-CoV-2 infections have occurred in this group. This study examines newborn care inequities for infants born to mothers with SARS-CoV-2.

**Methods:**

This study was conducted at Maine Medical Center in Portland, the largest hospital in Maine. Maternal SARS-CoV-2 infections from March 15, 2020 through April 1, 2021 were identified by PCR near time of delivery. Cases were matched to uninfected women by date of delivery. Chart review was conducted assessing demographic and clinical characteristics, comparing SARS-CoV-2 exposed and unexposed infants. The subset of SARS-CoV-2 exposed infants was further analyzed for trends in care by race. Protocol was exempt by MaineHealth IRB.

**Results:**

Twenty four women and their infants were identified with maternal positive SARS-CoV-2 PCR just prior to delivery. An additional 24 unexposed infants were enrolled. When compared to unexposed infants, SARS-CoV-2 exposed were more likely to be racial minorities (63% vs 21%, p = 0.003), to have foreign-born mothers (58% vs 0.4%, p< 0.05) or to receive health care in a language other than English (29% vs 0.4%, p =0.02). For infants born to SARS-CoV-2 infected mothers, only 29% had initial follow up visit in person with their primary care provider (13% of BIPOC infants vs 56% of non-BIPOC infants, p = 0.03). Time to in-person follow up for exposed infants varied by race, with median time of 21 days (range 2-53 days) for racial minorities and 7.5 days (range 2-30 days) for non minorities. All families were discharged with a thermometer and scale for home management. No infants required re-admission during the month after discharge. One exposed infant tested positive for SARS-CoV-2.

**Conclusion:**

The American Academy of Pediatrics recommends evaluation of newborns 3-5 days after discharge to identify maternal and child health factors affecting newborn well-being. The SARS-CoV-2 pandemic has made this challenging for patients, particularly for racial minorities. BIPOC pediatric patients were disproportionately affected by the pandemic in Maine, and were disproportionately affected by care discrepancies even when the infant was uninfected.

**Disclosures:**

**All Authors**: No reported disclosures

